# Novel human microbe-disease associations inference based on network consistency projection

**DOI:** 10.1038/s41598-018-26448-8

**Published:** 2018-05-23

**Authors:** Shuai Zou, Jingpu Zhang, Zuping Zhang

**Affiliations:** School of Information Science and Engineering, Central South University, Changsha, 410083 China

## Abstract

Increasing evidence shows that microbes are closely related to various human diseases. Obtaining a comprehensive and detailed understanding of the relationships between microbes and diseases would not only be beneficial to disease prevention, diagnosis and prognosis, but also would lead to the discovery of new drugs. However, because of a lack of data, little effort has been made to predict novel microbe-disease associations. To date, few methods have been proposed to solve the problem. In this study, we developed a new computational model based on network consistency projection to infer novel human microbe-disease associations (NCPHMDA) by integrating Gaussian interaction profile kernel similarity of microbes and diseases, and symptom-based disease similarity. NCPHMDA is a non-parametric and global network based model that combines microbe space projection and disease space projection to achieve the final prediction. Experimental results demonstrated that the integrated space projection of microbes and diseases, and symptom-based disease similarity played roles in the model performance. Cross validation frameworks and case studies further illustrated the superior predictive performance over other methods.

## Introduction

Joshua Lederberg systematically explained the concept of the microbiome for the first time as “Microbiome signifies the ecological community of commensal, symbiotic, and pathogenic microorganisms that literally share our body space”^1^. Many microorganisms inhabit the human body, comprising mainly bacteria, archaea, viruses, fungi and protozoa^2^. The number of bacteria inhabiting the human body is at least 10 times more than the number of human cells^3^. Thus, nearly 90% of the cells in the human body are microbial cells. These microbes exist in different organs of the human body, such as the gastrointestinal tract, respiratory tract, mouth, and skin^4^. Over the past few decades, there has been increasing interest in microbes that inhabit the human body^5^. 16S rRNA gene sequencing is generally used to study these microbes^6–9^. Moreover, the Human Microbiome Project (HMP) has successfully described the microbes in terms of their structure, function and diversity^10^; its goal is to generate a comprehensive catalogue of human associated microbes^11^.

The relationship between the microbiome and the host is complex. The microbiome inhabiting the human body can do both good and harm to the host. On the one hand, the microbiome is conducive to developing the immune system^12,13^, maintaining homeostasis^14^, protecting against pathogens, and drug metabolism^15^. On the other hand, there is strong evidence that microbes are associated with various diseases, such as obesity^16^, diabetes^17,18^, asthma^19^, and cancer^20^. Therefore, a comprehensive and detailed understanding of the relationships between microbes and diseases would be not only beneficial for disease prevention, diagnosis and prognosis, but would also promote the discovery of new drugs.

Currently, certain computational methods have been proposed to study microbes and human diseases^21,22^. These studies aimed to predict the impact of microbes on biological events and to identify functional subnetworks in microbiome-related diseases. However, because of a lack of data, little effort has been made to study the relationships between microbes and diseases. In 2016, Ma *et al*. established the first Human Microbe-Disease Association Database (HMDAD) using large-scale text mining, which provides experimental data for the study of microbe-disease associations. On the basis of the database, Chen *et al*. developed a method called KATZ measure for Human Microbe-Disease Association prediction (KATZHMDA)^23^, while Huang *et al*. proposed a method called Path-Based Human Microbe-Disease Association prediction (PBHMDA)^24^. Both methods achieve satisfactory predictive results. However, the microbes in HMDAD belong to different taxonomic levels, such as phylum, class, genus and species. An entire phylum or class contains thousands of individual species; therefore using an entire phylum or class to predict novel associations is inaccurate, which would lead to optimistic claims of the model performance. Hence, to improve the reliability and accuracy of experimental data, we reprocessed the data in HMDAD and kept the known microbe-disease associations at defined taxonomic levels: genus level and species level, respectively.

In this study, we developed a computational model based on network consistency projection to infer novel human microbe-disease associations (NCPHMDA) by integrating Gaussian interaction profile kernel similarity of microbes and diseases, and symptom-based disease similarity. The most significant difference from previous models is that our model is more simple and effective without any parameters. Additionally, symptom-based disease similarity is introduced to predict human microbe-disease association for the first time. To evaluate the prediction performance of NCPHMDA, cross validation frameworks (leave-one-out and 5-fold cross validation) were implemented on two datasets: genus level dataset and species level dataset, respectively. The experimental results illustrated that symptom-based disease similarity and integrated space projection have effects on the prediction performance of the model. Moreover, the results also demonstrated that our model has a favourable advantage over the other two state-of-the-art models. Furthermore, case studies of asthma and type 2 diabetes were implemented to evaluate the predictive performance of our model. Seven and eight of the top 10 predictions for these two diseases have been confirmed by recent research, respectively. Both cross validation frameworks and case studies fully demonstrated the powerful ability of NCPHMDA to predict novel microbe-disease associations.

## Results

### Construction and analysis of the microbe-disease association networks

**Figure 1:**
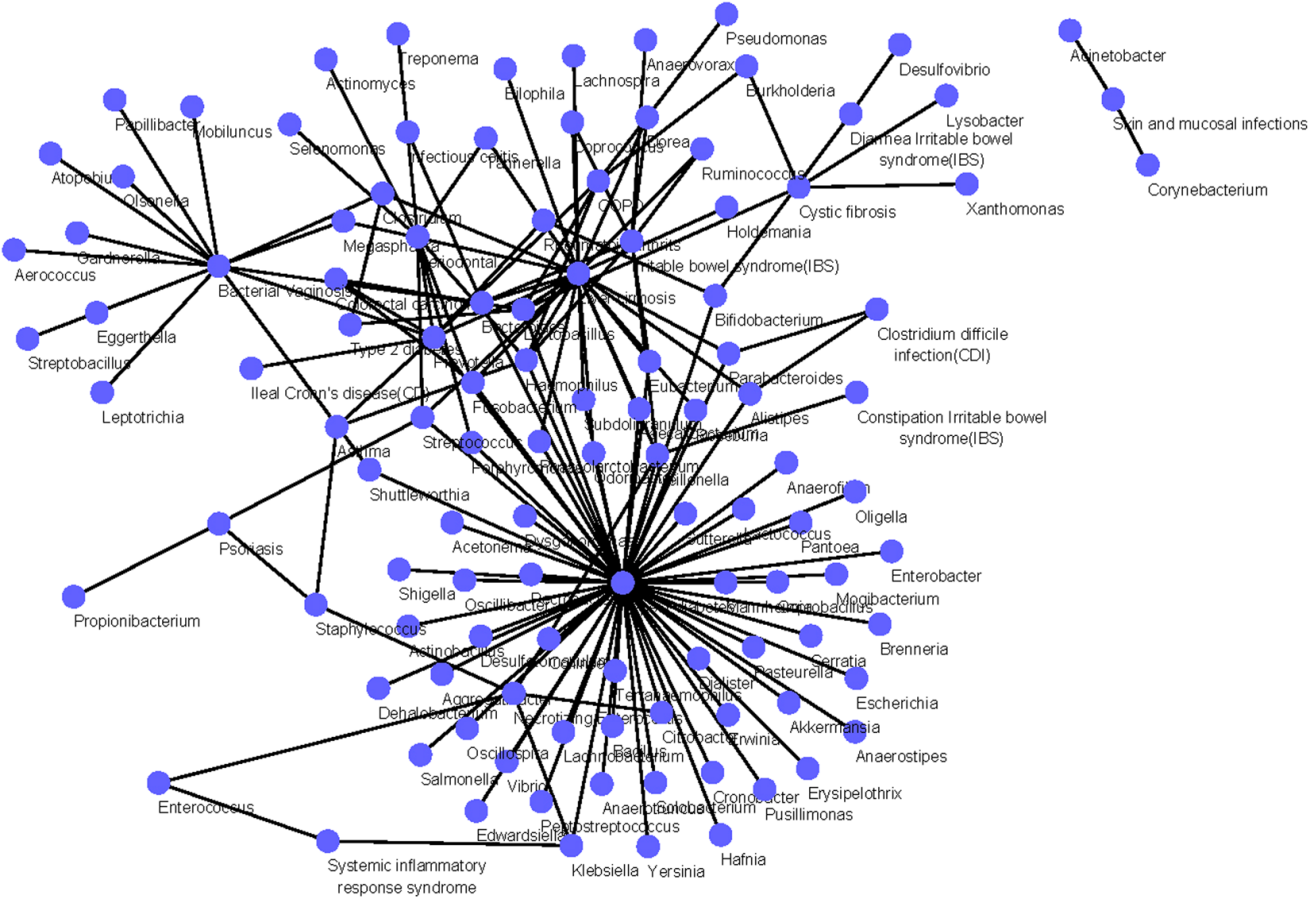
The microbe-disease association network of genus level.

The genus level dataset includes 155 known microbe-disease associations between 94 microbes and 20 diseases, while the species level dataset contains 180 known microbe-disease associations between 147 microbes and 30 diseases. Based on these two datasets, we constructed two different microbe-disease association networks. The association network of genus level can be seen in Fig. 1, and the association network of species level is shown in Supplementary Information S1. In each heterogeneous network, the nodes denote either microbes or diseases, and the edges correspond to the associations between microbes and diseases^25^.

**Figure 2:**
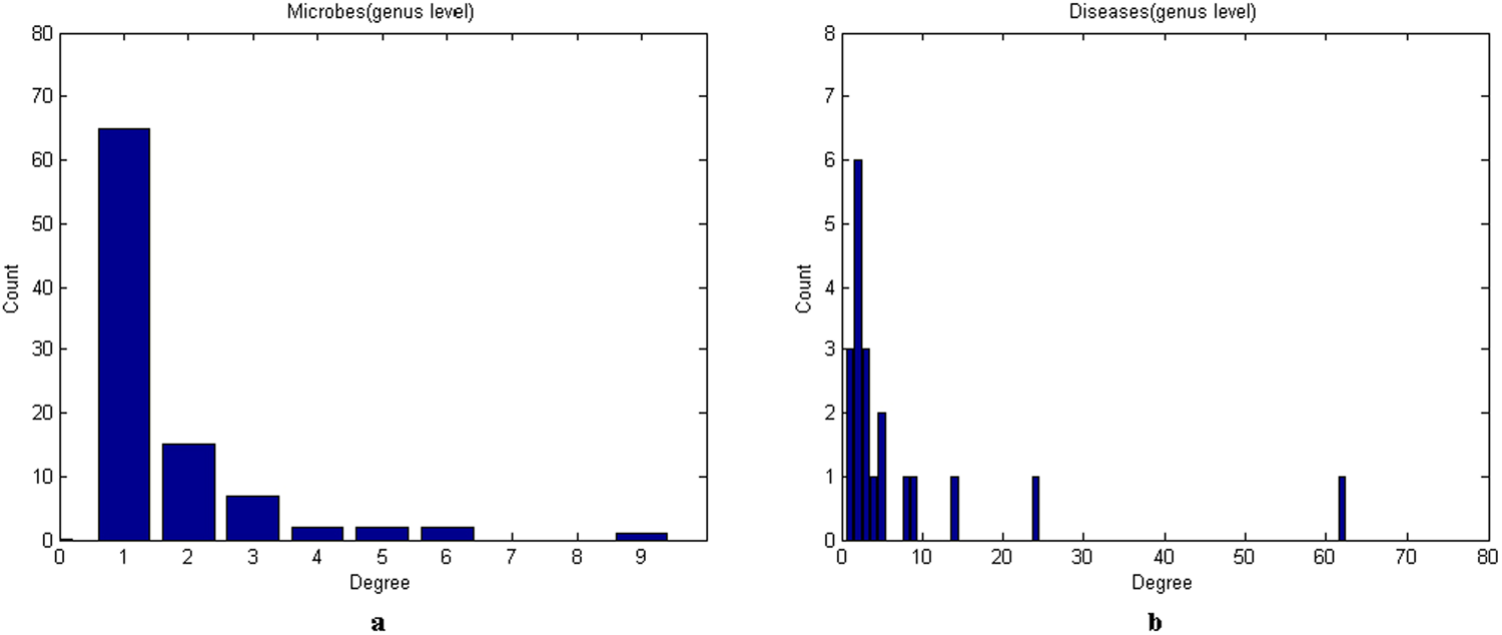
Degree distribution for microbes and diseases in the microbe-disease association network of genus level. (**a**) Degree distribution of microbes. (**b**) Degree distribution of diseases.

To obtain a comprehensive view of these two microbe-disease association networks, we further analysed some of their statistical characteristics^26^ (Table 1). The degree distributions of microbes and diseases in the microbe-disease association networks of genus level and species level are illustrated in Fig. 2 and Supplementary Information S2, respectively. The degree of a microbe node is the number of diseases associated with the given microbe. The degree of a disease node is defined similarly. On average, each microbe is associated with 1.65 and 1.22 diseases, while each disease is associated with 7.75 and 6 microbes in the microbe-disease association networks of genus level and species level, respectively.

**Table 1:** Global characteristics of the microbe-disease association networks.

Level	No.of microbes	No.of diseases	No.of microbe-disease associations	Average degree of microbes	Average degree of diseases
genus	94	20	155	1.65	7.75
species	147	30	180	1.22	6

### Performance evaluation

In this study, leave-one-out cross validation (LOOCV) and 5-fold cross validation (5-fold CV) were implemented on the known microbe-disease associations to evaluate the predictive performance of NCPHMDA. In each trail of LOOCV, every known microbe-disease association was left out as test samples, while the remaining associations were taken as training samples. It should be noted that the microbe similarity and disease similarity should be recalculated in each trail. For each disease, the microbes that do not have known associations with the given disease were considered as candidate microbes. The scores of all microbe-disease pairs could be obtained by implementing NCPHMDA. We then acquired the rank of all candidate microbes. The test samples that received higher ranks than a given threshold could be regarded as correct predictions. In 5-fold CV, the known microbe-disease associations were divided randomly and equally into five subsets. For each trial, one subset was processed as test samples and the other four subsets were processed as training samples. Moreover, receiver-operating characteristic (ROC) curves were implemented by plotting the true positive rate (TPR, sensitivity) against the false positive rate (FPR, 1-specificity) at different thresholds to check the performance of the model. Sensitivity refers to the percentage of test samples that ranked higher than the given threshold, while specificity means the percentage of test samples that ranked lower than the given threshold. The area under the ROC curve (AUC) can thus be calculated to reflect the predictive performance, where an AUC value of 1 indicates perfect performance, and 0.5 indicates random performance. As a result, when using genus level dataset, our method achieved AUC values of 0.9129 based on LOOCV and 0.9108 based on 5-fold CV. When using species level dataset, our method achieved AUC values of 0.9748 based on LOOCV and 0.9782 based on 5-fold CV. These results indicated a reliable and effective predictive performance.

**Figure 3:**
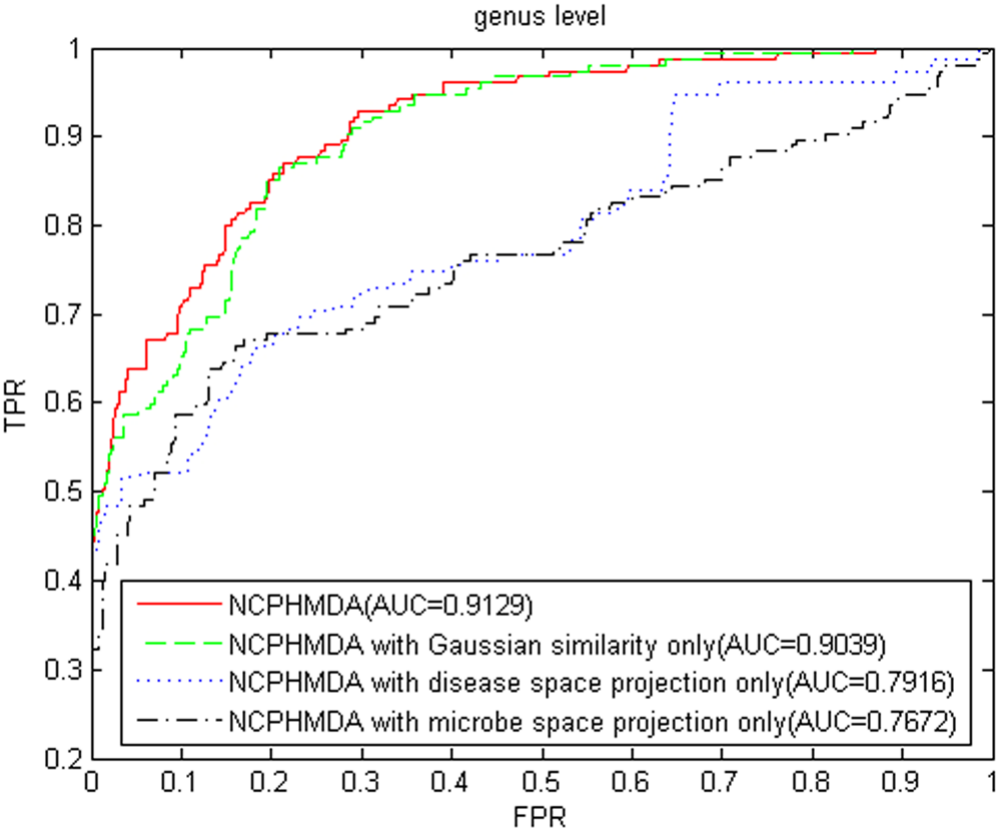
The ROC curves and AUC values of NCPHMDA based on LOOCV in different situations (genus level). (**a**) NCPHMDA with all information, (**b**) NCPHMDA with Gaussian interaction profile kernel similarity only, (**c**) NCPHMDA with disease space projection only, (**d**) NCPHMDA with microbe space projection only.

To evaluate the effectiveness of NCPHMDA, we tested its performance in different situations based on LOOCV. The results can be seen in Fig. 3 and Supplementary Information S3. Taking the experiment results of genus level for instance (Fig. 3), when using only Gaussian interaction profile kernel similarity, the AUC value of NCPHMDA decreased to 0.9039. This showed that integrating symptom-based disease similarity is conducive to improving the predictive performance of NCPHMDA. In addition, NCPHMDA achieved AUC values of 0.7916 and 0.7672 in disease space projection and microbe space projection, respectively. It demonstrated that integrated space projection could contribute to improve predictive performance.

### Comparison with other methods

As far as we know, KATZHMDA and PBHMDA are the state-of-the-art computational models for microbe-disease association prediction. KATZHMDA calculates correlations between nodes in the heterogeneous network to predict links, which was initially proposed to solve the friend prediction problem in a social network. PBHMDA is a path-based method that applies a special depth-first search algorithm to traverse all paths between microbes and diseases. The similarities of these two methods are: they are both achieved based on a heterogeneous network, which is constructed by connecting a microbe similarity network and a disease similarity network via the known microbe-disease associations; moreover, Gaussian interaction profile kernel similarity is applied to measure microbe similarity and disease similarity in these two methods. Importantly, in NCPHMDA, besides Gaussian interaction profile kernel similarity, symptom-based disease similarity was also introduced to measure disease similarity, which could improve the predictive performance. In addition, NCPHMDA does not need any parameters, which simplifies the model and improves the computational efficiency. Moreover, NCPHMDA is still applicable in situations where there are very few known microbe-disease associations.

**Figure 4:**
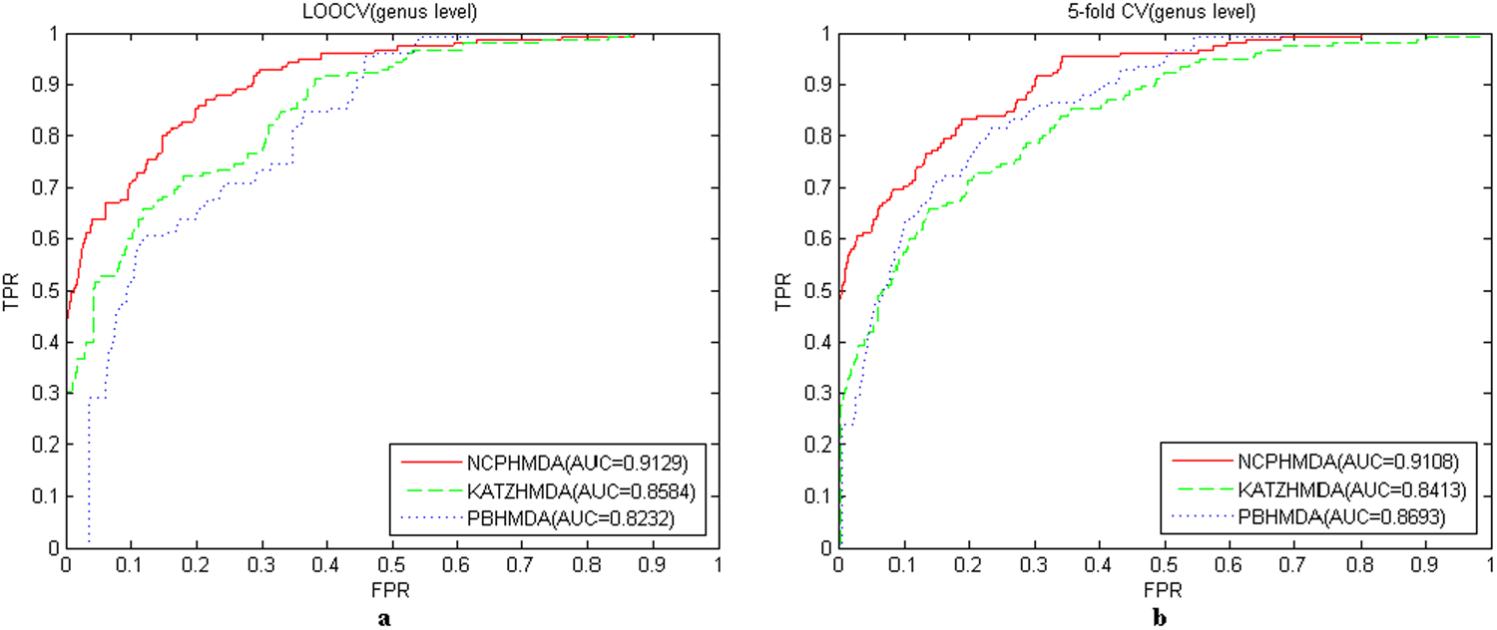
The ROC curves and AUC values of NCPHMDA, KATZHMDA and PBHMDA based on LOOCV and 5-fold CV (genus level). (**a**) The ROC curves and AUC values of NCPHMDA, KATZHMDA and PBHMDA based on LOOCV, (**b**) The ROC curves and AUC values of NCPHMDA, KATZHMDA and PBHMDA based on 5-fold CV.

To further evaluate the predictive performance of NCPHMDA, using the same parameters and datasets, we compare KATZHMDA and PBHMDA with NCPHMDA based on LOOCV and 5-fold CV. The results can be seen in Fig. 4 and Supplementary Information S4. Here, we also take the results of genus level for instance (Fig. 4): in LOOCV, NCPHMDA achieved a superior performance among all the methods, with an AUC value of 0.9129, while KATZHMDA and PBHMDA yielded AUC values of 0.8584 and 0.8232, respectively; in 5-fold CV, NCPHMDA still performed well, with an AUC value of 0.9108, which was significantly better than the performance of KATZHMDA and PBHMDA, with the AUC values of 0.8394 and 0.8693, respectively.

### CaseStudies

To illustrate the application of NCPHMDA to infer novel microbe-disease associations, we performed case studies of asthma and type 2 diabetes. The microbe-disease pairs that were not known to be associated in the dataset were the candidate microbe-disease associations. For each disease, the candidate microbes were ranked according to the prediction scores calculated by NCPHMDA. We used the top 10 that have been confirmed to date as the indicator to measure the predictive performance.

Asthma is a common long-term inflammatory disease of the airways of the lungs, which over the past few decades has increased noticeably in prevalence. Increasing studies showed that microbes might play important roles in the causation and exacerbation of asthma, hence in the co-morbidities due to asthma^27^. In the predicted list of asthma, seven microbes (genus level) ranking in the top 10 have been validated (Table 2). It has been found that *Bacteriodes* could be used as an early indicator of asthma later in life^24^. *Veillonella* is less represented in asthmatic patients^24^. A study demonstrated that the decrease of *Lactobacillus* could do help to prevent asthma^24^. Orally administered probiotic strain *Bifidobacterium* has a positive effect on atopic asthma^28^. Compared to healthy controls, there is a high level of *Fusobacterium* in asthmatic patients^24^. Research found that severe asthmatics are enriched in several taxa, with the largest fold-difference seen in a *Klebsiella*^29^. *Streptococcus* was found to be associated with pediatric asthma and allergic asthma^30^. To date, no relevant study has found that *Enterococcus*, *Desulfovibrio* and *Burkholderia* are related to asthma, but they could be considered as potential asthma-causing microbes.

**Table 2:** Prediction of the top 10 microbes (genus level) associated with asthma.

Rank	Microbe	Evidence
1	*Bacteroides*	PMID: 28275370^24^, PMID: 18822123^42^, PMID: 29161087^43^
2	*Enterococcus*	unconfirmed
3	*Veillonella*	PMID: 28275370^24^, PMID: 25329665^44^, PMID: 26424567^45^
4	*Lactobacillus*	PMID: 28275370^24^, Gutkowski *et al*.^28^
5	*Bifidobacterium*	Gutkowski *et al*.^28^, PMID: 26840903^46^
6	*Fusobacterium*	PMID: 28275370^24^, Dang *et al*.^19^, PMID: 27838347^47^
7	*Klebsiella*	PMID: 26220531^29^
8	*Desulfovibrio*	unconfirmed
9	*Streptococcus*	PMID: 28625914^30^, PMID: 27726947^48^
10	*Burkholderia*	unconfirmed

Type 2 diabetes is a long-term metabolic disorder that is characterized by high blood sugar, insulin resistance, and relative lack of insulin. Until 2015, there were nearly 392 million people diagnosed with type 2 diabetes. In the predicted list of type 2 diabetes, eight microbes (species level) ranking in the top 10 have been validated (Table 3). It has been found that *Helicobacter pylori* infection is higher in diabetic obese patients than non-diabetic subjects^31^. *Clostridum difficile* infection is increasingly seen among hospitalised patients with type 2 diabetes^32^. A study showed that *Staphylococcus aureus* plays a role in the development of type 2 diabetes^33^. The level of *Collinsella aerofaciens* in type 2 diabetes group is significantly lower than those in normal glucose tolerance group^34^. It has been confirmed that *Bacteroides vulgatus* and *Prevotella copri* species are associated with the development of type 2 diabetes^35^. Glycemic level in diabetes is affected by the persistence of *Porphyromonas gingivalis*^36^. High prevalence of *Escherichia coli* in diabetes patients would result in high mortality^37^. Although *Tropheryma whipplei* and *Bacteroides uniformis* have not been proved to be associated with type 2 diabetes, their presence in the top 10 predicted list could provide direction for future research.

**Table 3:** Prediction of the top 10 microbes (species level) associated with type 2 diabetes.

Rank	Microbe	Evidence
1	*Helicobacter pylori*	PMID: 18080918^31^, Li *et al*.^49^, Devrajani *et al*.^50^
2	*Clostridium difficile*	PMID: 23734349^32^, PMID: 27321318^51^, PMID: 21349600^52^
3	*Staphylococcus aureus*	PMID: 26439811^33^, PMID: 29024614^53^, PMID: 16495627^54^
4	*Collinsella aerofaciens*	Xiong *et al*.^34^
5	*Bacteroides vulgatus*	PMID: 28966614^35^
6	*Porphyromonas gingivalis*	PMID: 18582336^36^, PMID: 26792183^55^, Quintero *et al*.^56^
7	*Prevotella copri*	PMID: 28966614^35^
8	*Tropheryma whipplei*	unconfirmed
9	*Bacteroides uniformis*	unconfirmed
10	*Escherichia coli*	Deraje *et al*.^37^, Wang *et al*.^57^, Ye *et al*.^58^

## Discussion

Microbes play an important part in human health and disease; therefore, it is imperative that we obtain a comprehensive and detailed understanding of microbe-disease associations, and then use this knowledge to promote disease prevention, diagnosis and prognosis.

In this study, to improve the reliability and accuracy of the experimental data, we first reprocessed the data in HMDAD by keeping the known microbe-disease associations at defined taxonomic levels, then we obtained two independent datasets named genus level dataset and species level dataset. Next, we developed a computational model based on network consistency projection to infer novel microbe-disease associations. NCPHMDA is a global based method that combines microbe space projection and disease space projection to obtain the final prediction results. The important differences from previous methods are as follows: symptom-based disease similarity is introduced to integrate with Gaussian interaction profile kernel similarity to construct disease similarity; in addition, our method does not acquire any parameters, which simplifies the model and reduces the computation time. Moreover, the method is still applicable in situations where there are very few verified microbe-disease associations. NCPHMDA and cross validation frameworks (LOOCV and 5-fold CV) were implemented on the above-mentioned two datasets, respectively. The experiment results demonstrated that integrated network consistency projection and symptom-based disease similarity played roles in the predictive performance. Additionally, our method was demonstrated to be superior compared with two other state-of-the-art methods. Case studies of asthma and type 2 diabetes were implemented to illustrate the favourable performance of NCPHMDA. Taken together, the results demonstrated that NCPHMDA could be utilized as an efficient and effective model to reveal novel microbe-disease associations.

Despite the favourable results, some limitations still exist in this model. Firstly, the number of known microbe-disease associations is relatively few, which would have a negative effect on the prediction results. Studies should aim to discover more verified microbe-disease associations to expand the size of the database. Secondly, Gaussian interaction profile kernel similarities of microbes and diseases are calculated based on the known microbe-disease associations, which may cause bias towards diseases with more associated microbes and microbes with more associated diseases. Different datasets of microbes and diseases need to be integrated to reduce this bias. Thirdly, as far as we know, there is no specific standard dataset of microbe similarity; therefore, the microbe similarity network was constructed only based on known microbe-disease associations. A reasonable method needs to be developed to measure microbe similarity, which could then be applied to microbe-disease association inference in a future study.

A similar method^38^ was published while our manuscript was under consideration for publication. It happened that we and Bao *et al*.^38^ adopted the same method network consistency projection to predict novel human microbe-disease associations. Nevertheless, there are several important differences between these two papers. Firstly, to improve the reliability and accuracy of experimental data, we reprocessed the data in HMDAD and kept the known microbe-disease associations at defined taxonomic levels: genus level and species level, respectively. Secondly, it was the first time in this paper that symptom-based disease similarity has been introduced to integrate with Gaussian interaction profile kernel similarity of diseases to obtain the final disease similarity. Thirdly, based on two different datasets, we constructed two different microbe-disease association networks and analysed some of their statistical characteristics. As a result, experiments on these two datasets showed that our method in this paper performed better than Bao *et al*.’s in both LOOCV and 5-fold CV.

## Methods

### Dataset

The human microbe-disease association data can be retrieved from the Human Microbe-Disease Association Database (HMDAD, http://www.cuilab.cn/hmdad), which has recorded 483 verified microbe-disease associations between 39 human diseases and 292 microbes. After removing repeated microbe-disease entries and keeping the microorganisms at defined taxonomic levels, we finally acquired 155 microbe-disease associations between 94 microbes and 20 diseases at the genus level and 180 microbe-disease associations between 147 microbes and 30 diseases at the species level. In this study, they were called genus level dataset and species level dataset, respectively.

Symptom-based disease similarity data were also downloaded from HMDAD, which are calculated based on the term co-occurrence of diseases and symptoms. After converting the symptom disease into the corresponding microbe disease, we finally obtained 141 symptom similarity scores between 25 human diseases. Accordingly, there were 44 symptom similarity scores between 13 diseases in genus level dataset and 101 symptom similarity scores between 21 diseases in species level dataset.

### Microbe similarity

In this study, based on the assumption that microbes that are associated with highly similar diseases tend to be more similar, Gaussian interaction profile kernel similarity was applied to measure similarities between microbes^23^. Firstly, we construct the adjacency matrix *A* of the microbe-disease association network. *A*(*i*, *j*) is 1, if a known association exists between disease *i* and microbe *j*; otherwise it is 0. We then defined the microbe interaction profile *m*(*j*), a binary vector denoting the presence or absence between microbe *j* with every disease. Actually, it is the *j*th column of the adjacency matrix *A*. As a result, Gaussian interaction profile kernel similarity between microbe *j* and microbe *k* can be calculated from their interaction profiles:

1 $$MS(j,k)=exp(-{\gamma }_{m}||m(j)-m(k)|{|}^{2})$$

2 $${\gamma }_{m}={\gamma }_{m}^{\text{'}}/(\frac{1}{nm}\sum _{j=1}^{nm}||m(j)|{|}^{2})$$

where *γ*_*m*_ is the kernel bandwidth, which can be calculated from a new bandwidth *γ*′_*m*_ by the average number of associations with diseases per microbe; and *nm* is the number of all microbes. Here, *γ*′_*m*_ is simply set to 1.

### Disease similarity

#### Symptom-based disease similarity

Symptom-based disease similarity was measured by the symptoms shown by one specific disease. The association between diseases and symptoms were quantified by term co-occurrence^39,40^. For each disease *i* and each symptom *m*, the quantitative strength of their association could be measured as:

3 $${w}_{i,m}={W}_{i,m}log\frac{N}{{n}_{m}}$$

where *w*_*i*,*m*_ is defined as the term frequency-inverse document frequency. *W*_*i*,*m*_ denotes the co-occurrence (number of disease *i* and symptom *m* appear together). *N* is the number of all diseases and *n*_*m*_ is the number of diseases appearing together with symptom *m*. Log(*N/n*_*m*_) decreases the weights of symptoms that are generally related to many diseases and increases the weights of symptoms that are specifically related to some diseases. Then, each disease *i* can be represented as a vector:

4 $${d}_{i}=({w}_{i,1},{w}_{i,2},\cdots ,{w}_{i,M})$$

where *M* is the number of all symptoms. Here, cosine similarity is applied to measure the symptom-based disease similarity between disease *i* and disease *n*:

5 $$SS(i,n)=\,\cos ({d}_{i},{d}_{n})=\frac{{\sum }_{m=1}^{M}{d}_{i,m}{d}_{n,m}}{\sqrt{{\sum }_{m=1}^{M}{d}_{i,m}^{2}}\sqrt{{\sum }_{m=1}^{M}{d}_{n,m}^{2}}}$$

The cosine similarity ranges from 0 to 1, where 0 denotes no shared symptoms between two diseases and 1 denotes these two diseases have identical symptoms.

#### Gaussian interaction profile kernel similarity for diseases

Similarly, based on the assumption that diseases that are associated with highly similar microbes tend to be more similar, the Gaussian interaction profile kernel similarity for diseases could be calculated. We still use the adjacency matrix *A* constructed above. Here, we defined the disease interaction profile *d*(*i*), a binary vector denoting the presence or absence between disease *i* with every microbe. Actually, it is the *i*th row of the adjacency matrix *A*. As a result, the Gaussian interaction profile kernel similarity between disease *i* and disease *n* could be calculated from their interaction profiles:

6 $$GS(i,n)=exp(-{\gamma }_{d}||d(i)-d(n)|{|}^{2})$$

7 $${\gamma }_{d}={\gamma }_{d}^{\text{'}}/(\frac{1}{nd}\,\sum _{i=1}^{nd}||d(i)|{|}^{2})$$

where *γ*_*d*_ is the kernel bandwidth, which can be calculated based on a new bandwidth *γ*′_*d*_ by the average number of associations with microbes per disease; and *nd* is the number of all diseases. Similarly, *γ*′_*d*_ is also set to 1.

#### Integrated disease similarity

Based on the symptom-based disease similarity and Gaussian interaction profile kernel similarity for diseases mentioned above, the integrated disease similarity could be constructed as follows:

8 $$DS(i,n)\{\begin{array}{l}SS(i,n),disease\,i\,and\,disease\,n\,has\,symptom\,based\,similarity\\ \,GS(i,n),otherwise\,\end{array}$$

where *DS*(*i*, *n*) is the integrated similarity between disease *i* and disease *n*; *SS*(*i*, *n*) is the symptom-based similarity between disease *i* and disease *n*; *GS*(*i*, *n*) is the Gaussian interaction profile kernel similarity between disease *i* and disease *n*.

### NCPHMDA

**Figure 5:**
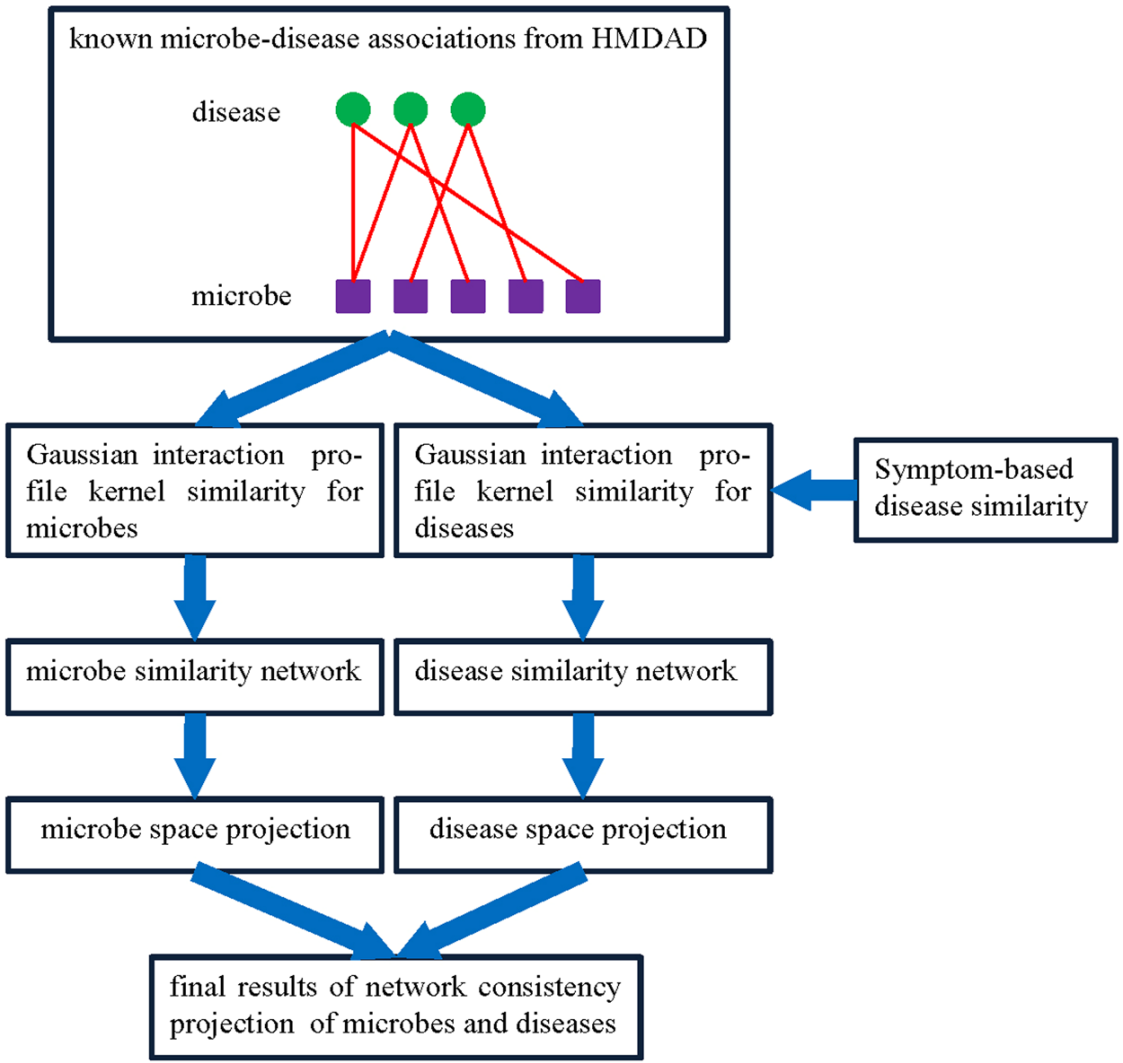
The flowchart of NCPHMDA.

In 2016, Gu *et al*. proposed a method called Network Consistency Projection for miRNA-Disease Associations (NCPMDA) to reveal the potential associations between miRNAs and diseases^41^. Inspired by its superior performance, in this study, we developed NCPHMDA to infer novel microbe-disease associations. The flowchart of NCPHMDA is shown in Fig. 5.

Network consistency means that the spatial similarity between microbe *j* associated microbes in the microbe similarity network and disease *i* associated microbes in the microbe-disease association network (or the spatial similarity between disease *i* associated diseases in the disease similarity network and microbe *j* associated diseases in the microbe-disease association network) is positively related to the association between disease *i* and microbe *j*. We projected the microbe similarity network and disease similarity network on the microbe-disease association network, respectively, and then combined these two space projections to obtain the final network consistency projection score. Vector space projection is applied to represent this process. Microbe space projection is defined as:

9 $$msp(i,j)=\frac{A{S}_{i}\times M{S}_{j}}{|A{S}_{i}|}$$

where *msp*(*i*, *j*) is the network consistency projection of *MS*_*j*_ on *AS*_*i*_. *AS*_*i*_ is the *i*th row of the microbe-disease association network; actually, it is the vector encoding the associations between disease *i* and all microbes. *MS*_*j*_ is the *j*th column of the microbe similarity network; actually, it is the vector denoting the similarities between microbe *j* and all microbes. |*AS*_*i*_| is the length of vector *AS*_*i*._ To avoid the denominator being 0, we use a small value *δ* instead of 0 in the adjacency matrix of the microbe-disease association network. Here, *δ* was set to 10^−30^.

Similarly, disease space projection can be defined as:

10 $$dsp(i,j)=\frac{D{S}_{i}\times A{S}_{j}}{|A{S}_{j}|}$$

where *dsp*(*i*, *j*) is the network consistency projection of *DS*_*i*_ on *AS*_*j*_. *DS*_*i*_ is the *i*th row of the disease similarity network; meanwhile, it is the vector denoting the similarities between disease *i* and all diseases. *AS*_*j*_ is the *j*th column of the microbe-disease association network; actually, it is the vector encoding the associations between microbe *j* and all diseases. |*AS*_*j*_| is the length of vector *AS*_*j*_.

Based on the microbe space projection and disease space projection calculated above, the final solution can thus be integrated as follows:

11 $$ncp(i,j)=\frac{dsp(i,j)+msp(i,j)}{|D{S}_{i}|+|M{S}_{j}|}$$

where *ncp*(*i*, *j*) is the final score of network consistency projection of disease *i* and microbe *j*. The code is available in Supplementary Information S5.

### Data availability

The dataset analyzed in the study is available in the Human Microbe-Disease Association Database (HMDAD), http://www.cuilab.cn/hmdad.

## Electronic supplementary material

Supplementary Information
