# Supplementary material for: Novel human microbe-disease associations inference based on network consistency projection
Source: Sci Rep. 2018 May 23;8:8034. doi: 10.1038/s41598-018-26448-8 (PMC5966389; doi:10.1038/s41598-018-26448-8)
Supplement: Supplementary file 1 — Supplementary Information [file 41598_2018_26448_MOESM1_ESM.pdf]

Novel human microbe-disease associations inference based on network consistency projection

Shuai Zou[1], **Jingpu Zhang**[1] **& Zuping Zhang**[1,*]

[1]School of Information Science and Engineering, Central South University, Changsha, 410083, China

* corresponding author's email: zpzhang@csu.edu.cn

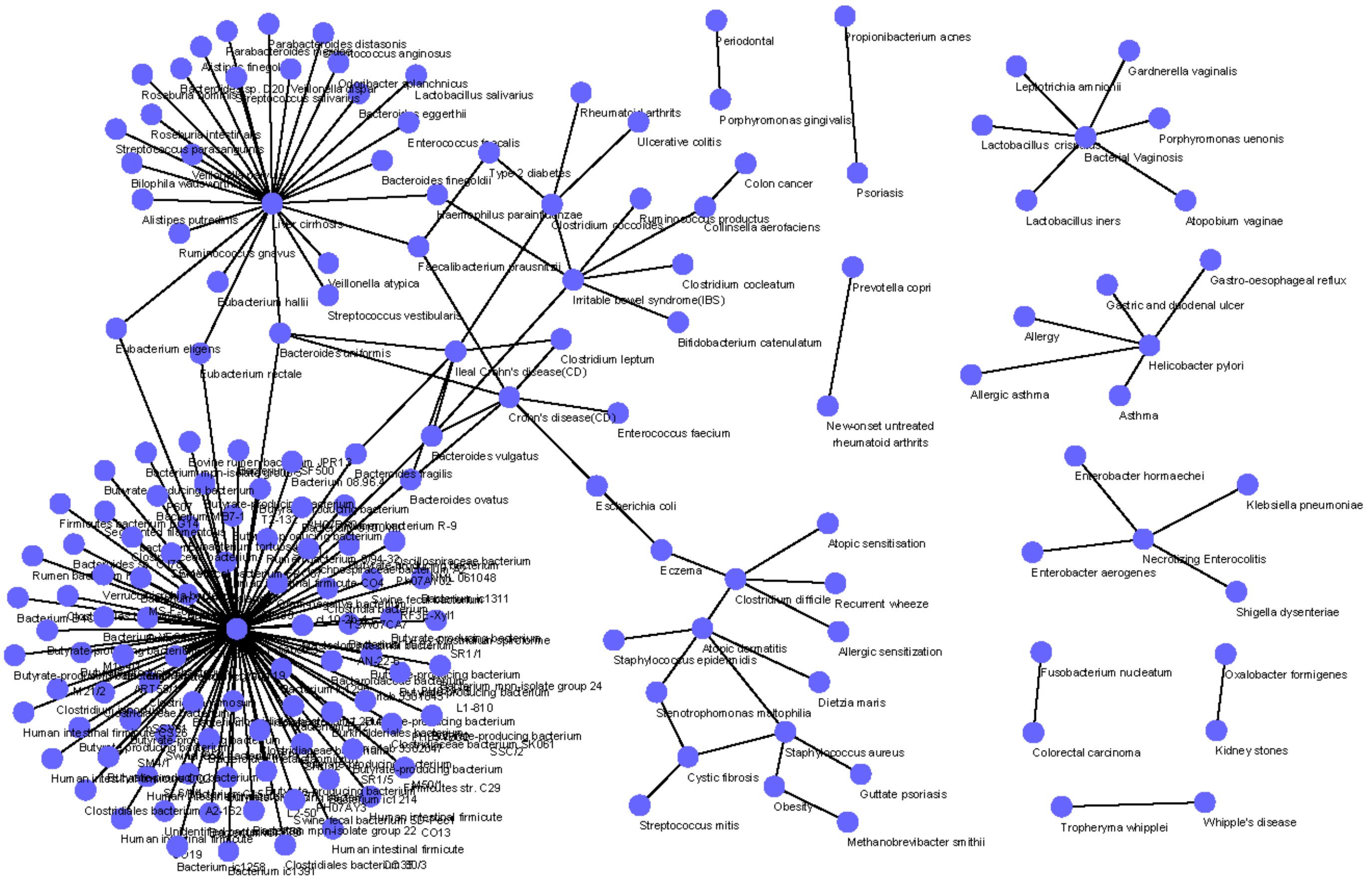

S1. The microbe-disease association network of species level.

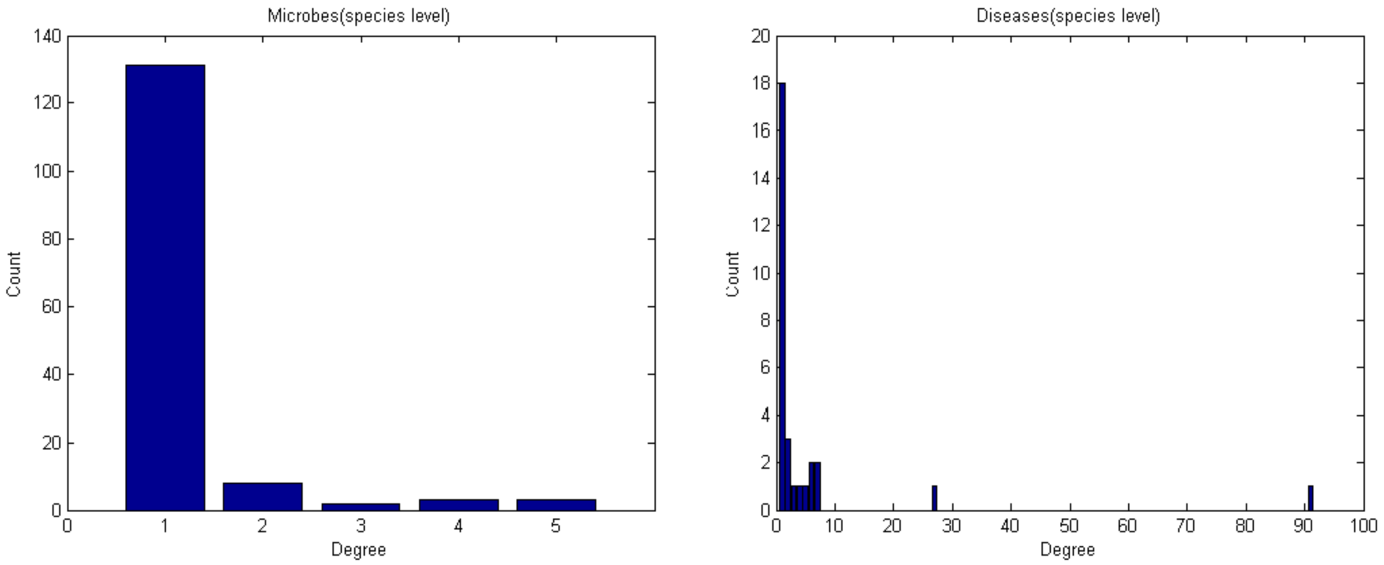

a **b**

S2. Degree distribution for microbes and diseases in the microbe-disease association network of species level. (a) Degree distribution of microbes. (b) Degree distribution of diseases.

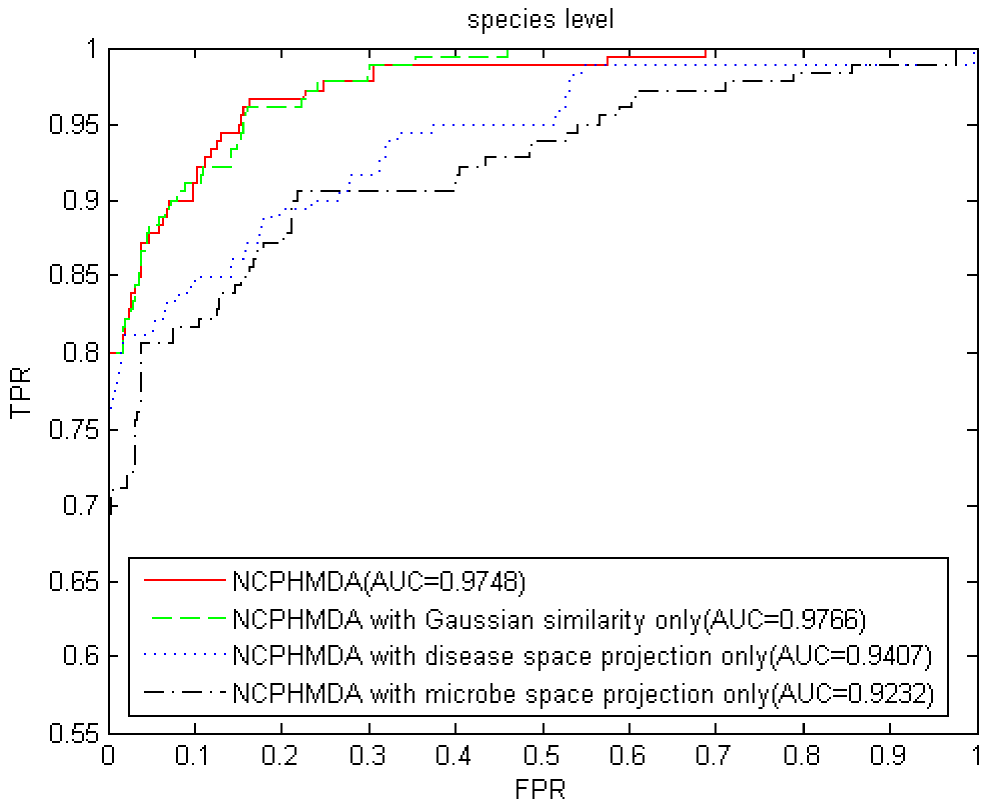

S3. The ROC curves and AUC values of NCPHMDA based on LOOCV in different situations (species level). (a) NCPHMDA with all information, (b) NCPHMDA with Gaussian interaction profile kernel similarity only, (c) NCPHMDA with disease space projection only, (d) NCPHMDA with microbe space projection only.

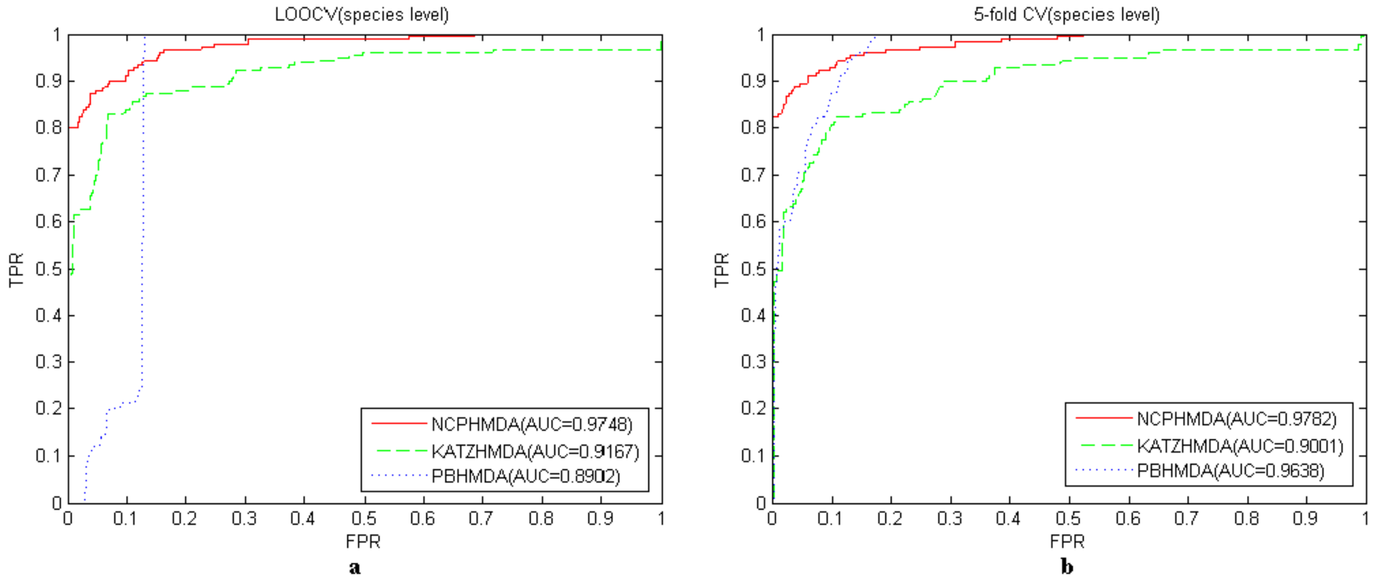

S4. The ROC curves and AUC values of NCPHMDA, KATZHMDA and PBHMDA based on LOOCV and 5-fold CV (species level). (a) The ROC curves and AUC values of NCPHMDA, KATZHMDA and PBHMDA based on LOOCV, (b) The ROC curves and AUC values of NCPHMDA, KATZHMDA and PBHMDA based on 5-fold CV.

S5. The code for NCPHMDA.

```
load microbesimilarity.mat
load diseasesimilarity.mat
load diseasesymptomsimilarity.mat
load diseasemicrobeinteraction.mat

[nd,nm]=size(diseasemicrobeinteraction);

for i=1:nd
    for j=1:nd
        if(diseasesymptomsimilarity(i,j))
            kd(i,j) = diseasesymptomsimilarity(i,j);
         else
            kd(i,j) = diseasesimilarity(i,j);
         end
    end
end

km=microbesimilarity;

ncp_d = zeros(nd,nm)+10^-30;
ncp_m = zeros(nd,nm)+10^-30;
ncp =zeros(nd,nm);

for i=1:nd
    ncp_m(i,:) = (diseasemicrobeinteraction(i,:)*km)/norm(diseasemicrobeinteraction(i,:));
end

for j=1:nm
    ncp_d(:,j) = (kd*diseasemicrobeinteraction(:,j))/norm(diseasemicrobeinteraction(:,j));
end

for i=1:nd
    for j=1:nm
        ncp(i,j) = (ncp_d(i,j) + ncp_m(i,j))/(norm(kd(i,:))+norm(km(:,j)));
    end
end

result=ncp';
```
